# Pathological differences in the bone healing processes between tooth extraction socket and femoral fracture

**DOI:** 10.1016/j.bonr.2022.101522

**Published:** 2022-03-24

**Authors:** Shinichirou Ito, Norio Kasahara, Kei Kitamura, Satoru Matsunaga, Toshihide Mizoguchi, Myo Win Htun, Yasuaki Shibata, Shinichi Abe, Masayuki Takano, Akira Yamaguchi

**Affiliations:** a Department of Oral and Maxillofacial Surgery, Tokyo Dental College, Tokyo, Japan; b Department of Histology and Developmental Biology, Tokyo Dental College, Tokyo, Japan; c Department of Anatomy, Tokyo Dental College, Tokyo, Japan; d Oral Health Science Center, Tokyo Dental College, Tokyo, Japan; e Tokyo Dental College Research Branding Project, Tokyo Dental College, Tokyo, Japan; f Department of Histology and Cell Biology, Nagasaki University Graduate School of Biomedical Sciences, Nagasaki, Japan

**Keywords:** Tooth extraction socket, Fracture, Bone regeneration, Healing process, Bone, Cartilage

## Abstract

Despite various reports on the bone healing processes of tooth extraction socket and long bone fracture, the differences of pathological changes during these healing processes remain elusive. This study aims to elucidate the underlying mechanisms behind the pathophysiology of bone regeneration between the tooth extraction socket and femoral fractures through a comparative study. Eight-week-old male mice were used in the experiments. The maxillary first molar was extracted, and intramedullary nailing femoral fracture (semistabilized fracture repair) was performed in the femur. Pathological changes in these bone injuries were investigated by micro-CT, histology, immunohistochemistry, and RT-PCR until day 7 post operation. Pathological changes in drill hole injury created in cortical bone of femur were also examined. Micro-CT analyses revealed increases in mineralized tissues in both the tooth extraction socket and femoral fracture. Histological examinations revealed that tooth socket was repaired by intramembranous ossification, and intramedullary nailing femoral fracture was healed by endochondral ossification. Immunohistochemical investigation revealed that tooth socket healing associated with Sp7-positive cells but not Sox9, aggrecan, and type II collagen, while femoral fracture models exhibited positive signals for all antibodies. RT-PCR analyses revealed the expression of *Sp7*, *Col1a1*, and *Col2a1* in tooth socket healing, and the expression of *Sp7*, *Col1a1*, *Runx2*, *Sox9*, *Acan*, *Col2a1*, and *Col10a1* in intramedullary nailing femoral fracture. Drill hole injury was repaired primarily by intramembranous ossification when the periosteum was removed before making the hole. The present study demonstrated that the absence of cartilage appearance during tooth extraction socket healing indicates it as distinctly different pathological features from the healing processes of semistabilized femoral fracture. This study contributes to the understanding of the molecular and cellular characteristics of bone healing among the different sites of bone injury.

## 1 Introduction

Bone regeneration is conducted by two modes of ossification: intramembranous or endochondral ossification. Regardless of the mode of ossification, bone healing is successfully achieved by the interaction of various factors including cell lineage, growth factor, revascularization, and mechanical loading (Claes et al., 2012; O'Keefe, 2015; Einhorn and Gerstenfeld, 2015). To understand these events, accurate pathological changes in various bone healing stages should be clarified in different modes of bone regeneration, because tooth extraction sockets are repaired by intramembranous ossification (Vieira et al., 2015), while some long bone fractures are healed by intramembranous or endochondral ossification (Claes et al., 2012; O'Keefe, 2015). It is important to determine the mechanisms by which the regulation of these two different modes of ossification occurs.

Despite various reports on tooth extraction socket healing (Arioka et al., 2021; Horibe et al., 2021; Vieira et al., 2015; Wang et al., 2020; Yi et al., 2021; Yuan et al., 2018; Zhang et al., 2020; Zhang et al., 2021), the comparative studies on pathological changes in bone regeneration between the tooth socket and long bones have not been conducted. Tooth extraction socket healing has a unique environment during bone regeneration; it is always surrounded by alveolar bone, and this might provide constant stability and retention of plasma clots during the healing process. In contrast, long bone fractures usually require a fixing device to acquire this stability of the fracture site. If fixation is unstable, long bone fractures are often repaired through endochondral ossification (Claes and Heigele, 1999). Comparative pathological studies of healing processes between tooth extraction sockets and long bone fractures provides a clue to the solution of why the extraction socket is always repaired by intramembranous ossification in the absence of cartilage formation.

Recent advances in cell lineage-tracing techniques provide a great opportunity to understand the lineages and fates of cells participating in skeletal tissue formation and regeneration (Debnath et al., 2018; Matsushita et al., 2020; Mizoguchi and Ono, 2021; Mizoguchi et al., 2014; Ransom et al., 2016; Shi et al., 2017). For example, Debnath et al. (2018) discovered that periosteal stem cells located in long bones and calvaria in mice could form bone by intramembranous ossification, whereas they acquired the capacity to undergo endochondral ossification depending on their plasticity. Recently, these techniques have been applied to determine the origin and fate of cells participating in tooth extraction socket healing (Arioka et al., 2021; Wang et al., 2020; Yi et al., 2021; Yuan et al., 2018; Zhang et al., 2020; Zhang et al., 2021). Although most of these studies used mice that had been developed primary for the analysis of skeletal tissue development and regeneration other than dental tissues, application of these mice is valuable for disclosing the lineage and fate of cells involved in tooth socket healing. To progress these studies, a precise investigation of the pathological changes in various healing processes between tooth extraction sockets and long bone fractures is necessary.

In the present study, we investigated the pathological changes at various healing stages in tooth extraction sockets and femoral fractures. This study provides a research platform for understanding the molecular and cellular characteristics of bone healing among the different sites of bone injury including tooth extraction socket.

## 2 Materials and methods

### 2.1 Experimental animals

All mice were maintained at room temperature under specific pathogen-free conditions. All experiments were performed according to the animal welfare guidelines and were approved by the Animal Care and Use Committee of Tokyo Dental College (No. 210101). Eight-week-old male C57BL6 mice were purchased from Sankyo Labo Service Corporation (Tokyo, Japan). Mice were intraperitoneally anesthetized with a mixture of medetomidine hydrochloride (0.75 mg/kg), midazolam (4.0 mg/kg), and butorphanol (5.0 mg/kg) for surgery. To collects tissue samples, mice were anesthetized with isoflurane, and euthanized by cervical spine dislocation.

### 2.2 Tooth extraction

The left maxillary first molar was carefully extracted using tweezers. After tooth extraction, maxillary bones were sampled at 3, 5, and 7 days, and subjected to micro-computed tomography (micro-CT) and histological analyses as described below.

### 2.3 Femoral fracture

We first applied the modified intramedullary nailing model of femur in accordance with a semistabilized fracture repair (Collier et al., 2020). Briefly, the center of the diaphysis was cut from above periosteum using a steel bar (0.8 mm diameter) under water cooling. Intramedullary fixation of the fractured femur was performed by inserting a 30 G needle (01-134; NIPRO, Osaka, Japan) from the distal end of the femur. Femora were collected at 3, 5, and 7 days after the fracture, and subjected to micro-CT and histological analyses. We also applied drill hole models with or without removal of the periosteum before making the drill hole. Drill hole models were performed as described previously (Matsushita et al., 2013). Briefly, a drill hole injury was created using a pin vise (74112; TAMIYA Inc., Shizuoka, Japan) with a diameter of 0.8 mm in the anterior position of the diaphysis of femur (approximately 7–8 mm from the distal end) with or without removal of the periosteum. Injured femur bone samples were collected on day 7 after drilling the drill holes. These samples were subjected to histological analysis.

### 2.4 Micro-CT analysis

**Fig. 1:**
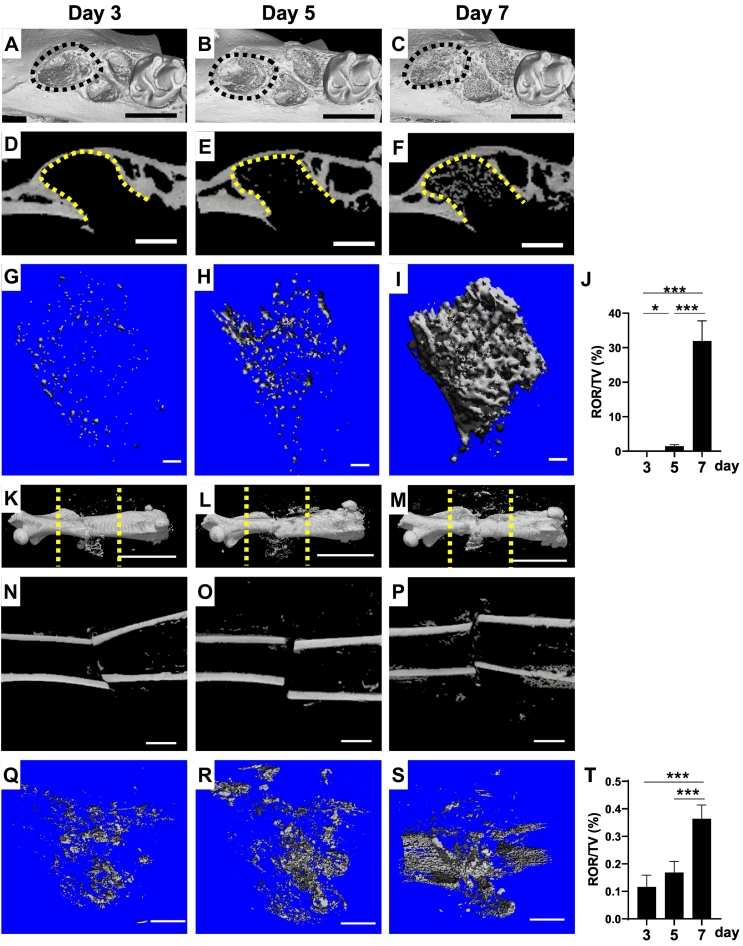
MicroCT images of tooth extraction sockets (A–I) and intramedullary nailing femoral fracture (K–S) on day 3, day 5, and day 7 after operation. Occlusal view of 3-D image including extraction sockets (black dot lines) (A–C). 2-D images of extraction sockets (yellow dot lines) (D–F). Structures of mineralized tissues extracted from 3-D images of tooth extraction sockets (G–I). Lateral view of 3-D image including femoral fractures (K–M). Radiopaque region (ROR) excluding cortical bone was measured between yellow lines. 2-D images of femoral fractures (N–P). Structures of mineralized tissues extracted from 3-D images of femoral fractures (Q–S). J and T are time course of changes in ROR/tissue volume (TV) at tooth extraction sockets (J) and femoral fracture (T), respectively. *: *P*<0.05, ***: *P*<0.001. n = 5. Scale bar = 1 mm (A–C, N–P, Q–S), 500 μm (D–F), 100 μm (G–I), and 5 mm (K–M).

Fixed samples were analyzed using a micro-CT 50 (Scanco Medical, Bruttisellen, Switzerland). Slice data were used to construct three-dimensional (3D) images using the volume-rendering technique using analytical software of TRI/3D-BON (Ratoc System Engineering, Tokyo, Japan) and VGSTUDIO MAX 3.5 (Volume Graphics GmbH, Heidelberg, Germany). The scanning parameters were as follows: 10 μm or 7.4 μm voxel size, 90 kV and 44 μA. The extraction socket and femur fractures were analyzed using software (Scanco Medical). The volume of radiopaque region (ROR) / total tissue volume (ROR / TV, %) formed in the extraction socket of the mesial root was measured using the software on days 3, 5, and 7 after the operation. In the cases of femoral fracture, the measurements were performed using the same software at an 8 mm ROI centered at the midpoint of the fracture, which comprised a total of 500 slices along axial plain (see yellow dot lines in Fig. 1K, L, and M).

### 2.5 Histological observation

The samples obtained from the maxilla and femur used for operations were fixed in 4% paraformaldehyde for 2 days and decalcified in 10% ethylenediaminetetraacetic acid solution (EDTA). After dehydration, the samples were embedded in paraffin. Serial sagittal sections were prepared from the maxillary samples. Serial frontal sections were prepared from femur samples. These sections were stained with hematoxylin-eosin (H-E) and Safranin O. They were also used for immunohistochemical analyses.

**Fig. 2:**
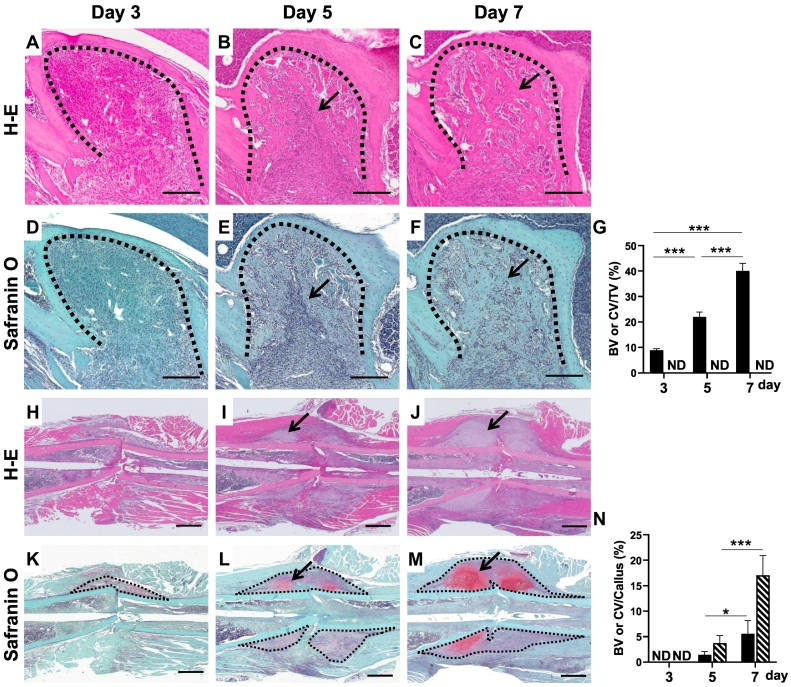
Low power histological features of tooth extraction socket (A–F) and intramedullary nailing femoral fracture (H–M) on day 3, day 5, and day 7 after operation. Newly formed bones increased in the tooth extraction sockets depending on days after operation (black arrows in B, C, E, and F). Cartilage formation area (black allows in I, J, L, and M) appeared during femoral fractures. Cartilage regions are red in Safranin O-stained sections (I, J, L, and M). Note that no Safranin O-stained cartilage during tooth socket healing (D–F). H-E stain (A–C, H–J) and Safranin O counterstained with hematoxylin (D–F, K–M). Black dot lines in A–F indicate border of tooth extraction socket and preexisting alveolar bone. Black dot lines in K, L, and M represent ROI to measure bone and cartilage area. Bone histomorphometric analysis of volume of newly formed bone and cartilage (G and N). G: Bone and cartilage volume in the tooth extraction socket, and those in the callus of femoral fracture (N). Closed bars indicate bone volume, and hatched bars indicate cartilage volume in G and N. *: *P*<0.05, ***: *P*<0.001. n = 5. Scale bar = 200 μm (A–F) and 1 mm (H–M).

The areas of bone and cartilage formation at the site of regeneration were quantified by measuring the bone volume (BV) and cartilage volume (CV) separately using the Image-Pro PLUS software (Media Cybernetics Inc., Rockville, USA) in the tooth extraction socket and femoral fracture, respectively. The ROI used to measure the amounts of bone and cartilage was defined as callus formed around the fracture sites excluding original cortical bone (see black dot lines in Fig. 2K–M, and yellow dot line in Fig. 6A). We applied two ROIs to measure BV and CV in drill hole mode; one is entire callus including cortical gap (see yellow dot lines in Fig. 6B, C) and another for only the cortical gap region (see red dot lines in Fig. 6B, C).

### 2.6 Immunohistochemical analysis and *in situ* hybridization

The expression of Sp7 (osterix), type II collagen, Sox9, and aggrecan (ACAN) was investigated by immunohistochemistry as described previously (Mitomo et al., 2019). The sections were treated with the primary antibodies using the following antibodies: anti-Sp7/osterix antibody (ab22552, Abcam, Cambridge, UK, diluted 1:1000), anti-Col2 antibody (ab34712, Abcam, diluted 1:400), anti-Sox9 antibody (ab5535, Millipore, Darmstadt, Germany, diluted 1:800), and anti-ACAN antibody (13880-1-AP, Proteintech, USA, diluted 1:500). After washing with PBS, the sections were incubated with a secondary antibody using EnVision+ Dual Link (DAKO, Santa Clara, CA USA). To detect immunoreaction, the sections were treated with ImmPACT DAB substrate (Funakoshi, Tokyo, Japan). After counterstaining with hematoxylin or light green, immunoreactions were observed under a microscope.

The mouse *Col2α1*-pBluescript KS(−) plasmid was kindly gifted by Dr. Toshihisa Komori (Nagasaki University, Japan). Labeling of probes with digoxigenin and *in situ* hybridization was performed as described previously (Shibata et al., 2004) with a slight modification. *In situ* hybridization for the detection of 28S rRNA with oligonucleotide probe was also performed for the confirmation of RNA retention and optimization of proteinase K concentration. The sense probe was used as negative control.

### 2.7 RNA isolation and quantitative real-time PCR

Since the tooth sockets of mouse upper molar tooth are very small, we used the left maxilla including extraction sockets for RNA isolation. In the semistabilized femoral fracture model, the whole femur including fracture site was subjected to RNA isolation as described previously (Mohan et al., 2005). Total RNA was isolated from these bones as previously described (Yang et al., 2017). The maxilla and femur samples were dissected on days 0, 3, 5, and 7 after the operation. Quantitative real-time PCR was performed by using the One-Step SYBR Prime Script PLUS RT-PCR (TAKARA, Shiga, Japan) using Step One Plus or 7500 Fast systems (Thermo Fisher Scientific). The mRNAs examined were *Sp7* (*Osterix*), *Col1a1* (*type I collagen*), *Runx2*, *Sox9*, *Acan* (*Aggrecan*), *Col2a1* (*type II collagen*), and *Col10a1* (*type X collagen*). Relative expression was determined using Glyceraldehyde-3-phosphate dehydrogenase (*GAPDH*) as an internal control. Expression levels were calculated as fold-change relative to control group (0 days), which were isolated immediately after the operations. The primers used for each gene are shown in Table 1 in Supplemental information.

### 2.8 Statistical analysis

Data were analyzed for normality using the Shapiro-Wilk test. Statistical analyses were performed using a one-way analysis of variance (ANOVA) multiple comparison test and Student's *t*-test with GraphPad Prism 8 (GraphPad Software, La Jolla, CA, USA). The results were expressed as mean ± standard deviation (SD), and statistical significance was set a *P* < 0.05.

## 3 Results

### 3.1 Micro-CT analyses

Low power magnification 3D images of extraction sockets revealed the increased irregular-shaped radiopaque regions (RORs) depending on the days after tooth extraction (Fig. 1A–C). On two-dimensional (2D) images of the extraction sockets, tiny spots of radiopaque structures appeared on day 5, and a substantial level of anastomosing radiopaque structure appeared on day 7 (Fig. 1E, F). Fig. 1G–I summarizes newly formed radiopaque structures in the extraction sockets that were reconstructed from the original 3D data shown in Fig. 1A–C. On day 3, a small amount of psammous RORs was observed in the extraction sockets (Fig. 1G). On day 5, the volume of RORs was increased by fusing adjacent structures (Fig. 1H). On day 7, a substantial volume of ROR showing plate-like structure anastomosing each other appeared (Fig. 1I). Quantitative data on ROR on each day were calculated from the 3D structure and are shown in Fig. 1J as ROR/TV. The volume increased until day 7.

Fig. 1K–M shows low-magnification images of femoral fractures on days 3, 5, and 7. A small number of radiopaque structures appeared around the fracture site on day 3 after the operation, and the distribution of these structures expanded around the fracture site depending on the days after the operation. On 2D images of the fracture site, ROR expanded along periosteal region depending on days after the operation (Fig. 1N–P). Fig. 1Q–S shows newly formed radiopaque structures around the fracture site that were reconstructed from the original 3D data shown in Fig. 1K–M. The distribution of the ROR expanded according to the number of days after fractures. Quantitative data concerning the volume of ROR around the fracture sites are summarized in Fig. 1T as ROR/TV. The volume increased until day 7.

### 3.2 Histological and immunohistochemical analyses of the tooth extraction sockets

**Fig. 3:**
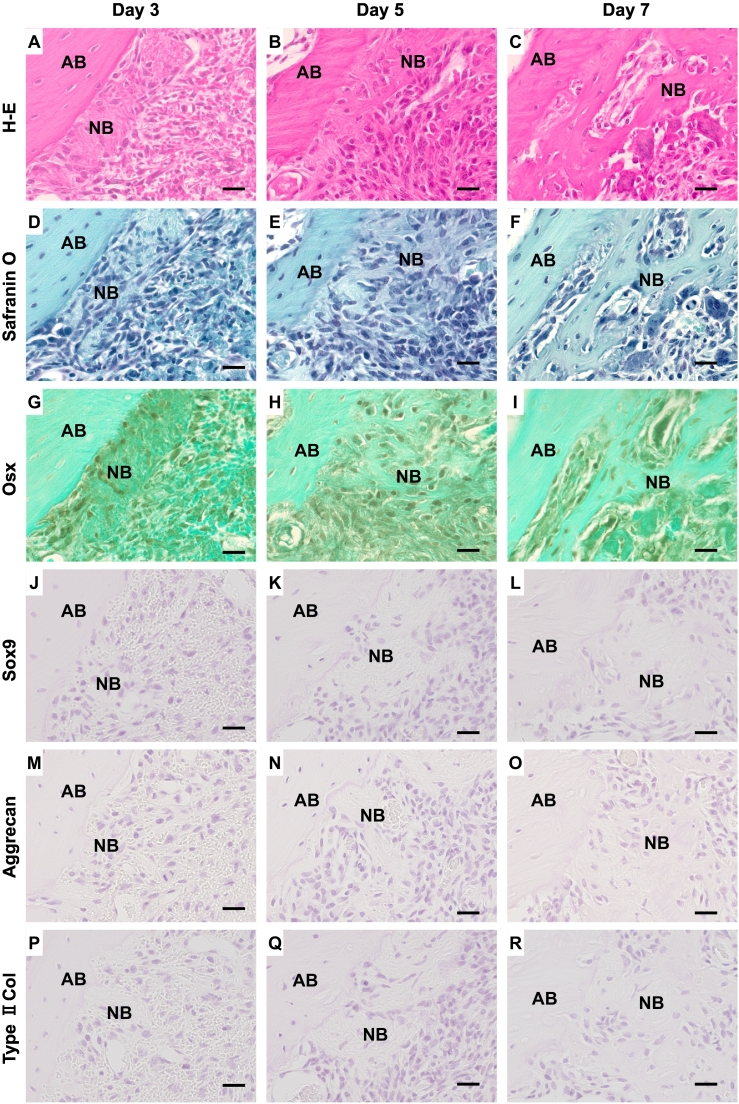
Immunohistochemical studies in tooth socket healing (G–R). Newly formed bones (NB) appeared adjacent to preexisting alveolar bone (AB) on day 3 after the operation. No cartilage matrix identified is seen during tooth socket healing (H-E stain: A–C, Safranin O stain: D–F). Immunostaining for osterix (Osx) (G–I), Sox9 (J–L), aggrecan (M–O), and type II collagen (Type II col) (P–R). Scale bar = 20 μm.

Mesenchymal cells associated with a small amount of immature bone were observed on the surface of the preexisting trabecular bone surface (Fig. 3A). Osterix-positive cells were scattered on the surface of the immature bone (Fig. 3G). On days 5 and 7, newly formed trabecular bones increased (Figs. 2B, E, 3B, E). Covering cells of the bone trabeculae were positive for osterix (Fig. 3I), and newly formed trabecular bones occupied almost region of the tooth sockets on day 7 (Figs. 2C, F, 3C, F). Osterix-positive cells were distributed on their bone surface (Fig. 3I). During the healing process of the tooth extraction sockets, no chondrocyte and cartilage matrices were identified by Safranin O staining (Figs. 2D–F, 3D–F). Immunohistochemical studies also showed no reactions to Sox9 (Fig. 3J–L), aggrecan (Fig. 3M–O), and type II collagen (Fig. 3P–R) throughout the healing process. The BV formed in the extraction sockets increased in a time dependent manner, whereas no cartilage appeared during healing process (Fig. 2G).

### 3.3 Histological and immunohistochemical analyses of the fracture repair

**Fig. 4:**
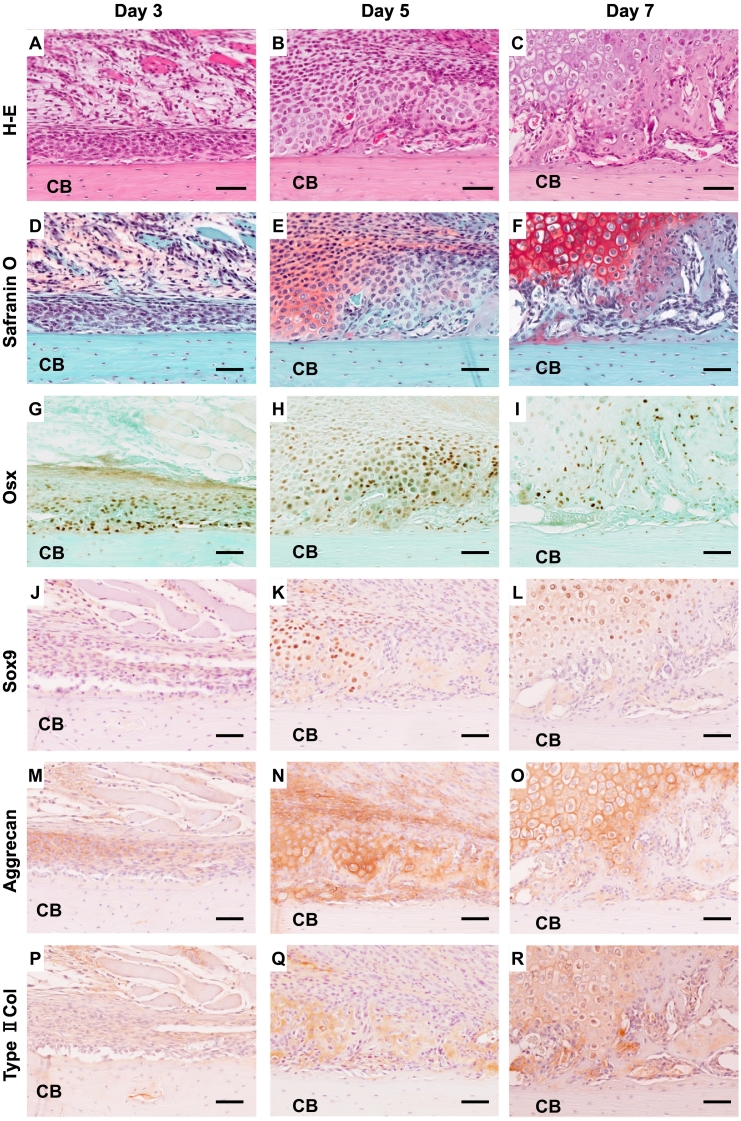
Immunohistochemical studies in intramedullary nailing femoral fracture (G–R). Newly formed bones and cartilage are seen on days 5 and 7 after the operation (H-E stain: A–C, Safranin O stain; D–F). Immunostaining for osterix (Osx) (G–I), Sox9 (J–L), aggrecan (M–O), and type II collagen (Type II col) (P–R). Scale bar = 50 μm.

On day 3 after fracture, numerous inflammatory cell infiltration associated with minute ectopic ossification were observed in the soft tissues around the fracture sites. Fibroblastic cells were arranged in several layers on the periosteal surface on day 3 (Fig. 4A, D). Numerous osterix-positive cells were observed among these cell layers, whereas no apparent bone formation was observed (Fig. 4G). No positive signals for cartilage-related proteins appeared on day 3 (Figs. 2K, 4D, J, M, P). On day 5, apparent bone trabeculae, which were surrounded by osterix-positive cells, appeared on the periosteal surface (Fig. 4B, E, H). Cartilaginous callus formation on the outer layer of the fracture site was recognized by Safranin O-stained sections (Figs. 2L, 4E). Numerous osterix-positive cells were observed in the newly formed trabecular bone region (Fig. 4H). Some small cells located in Safranin O-positive region were positive for osterix (Fig. 4E, H). Cells located in Safranin O-positive area were Sox9 positive (Fig. 4K), and extracellular matrices in these areas were positive for aggrecan and type II collagen (Fig. 4N, Q). On day 7, large cartilaginous calluses were formed around the fracture site (Fig. 2J, M). More mature trabecular bones were observed at the edge of the callus, and hypertrophic cartilage covered these bone trabeculae (Fig. 4C, F). Osterix-positive cells were scattered on the trabecular bone surface and cartilaginous area (Fig. 4I). Almost all chondrocytes in the Safranin O-positive area were Sox9 positive, and the extracellular matrices in this area were positive for aggrecan and type II collagen (Fig. 4F, L, O, R). The volumes of bone and cartilage increased in time-dependent manner (Fig. 2N).

### 3.4 Expression of osteoblast and chondrocyte related mRNA during healing process of tooth socket and femoral fracture

**Fig. 5:**
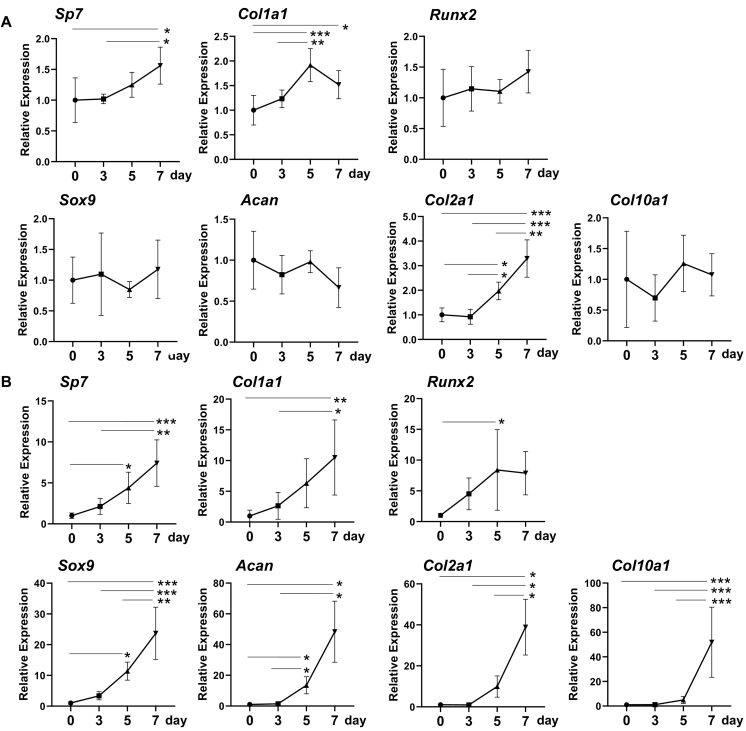
Time course of changes in mRNA expression in tooth socket healing (A) and intramedullary nailing femoral fracture (B). *: *P*<0.05, **: *P*<0.01, ***: *P*<0.001. n = 5.

Fig. 5 summarizes the time course of changes in mRNA expression in tooth socket (A) and femoral fracture (B). During tooth extraction healing, *Sp7* mRNA expression significantly increased on day 7 compared to that on day 0. *Col1a1* mRNA expression significantly elevated only day 5, compared with that on day 0. *Col2a1* expression was significantly increased on days 5 and 7, compared to that on day 0 (Fig. 5A). *In situ* hybridization revealed that a positive signal for *Col2a1* could be detected in some mesenchymal cells (Supplemental Fig. 1). No significant increases were observed in *Runx2*, *Sox9*, *Acan*, and *Col10a1* mRNA during tooth socket regeneration (Fig. 5A). During femoral fractures, *Sp7* mRNA was significantly elevated on days 5 and 7, *Col1a1* mRNA on day 7, and *Runx2* on day 5, compared with those in on day 0 (Fig. 5B). Among chondrocyte-related mRNAs, *Sox 9*, *Acan*, *Col2a1*, and *Col10a1* increased in a time dependent manner.

### 3.5 Histological comparison of the three fracture models of femur

**Fig. 6:**
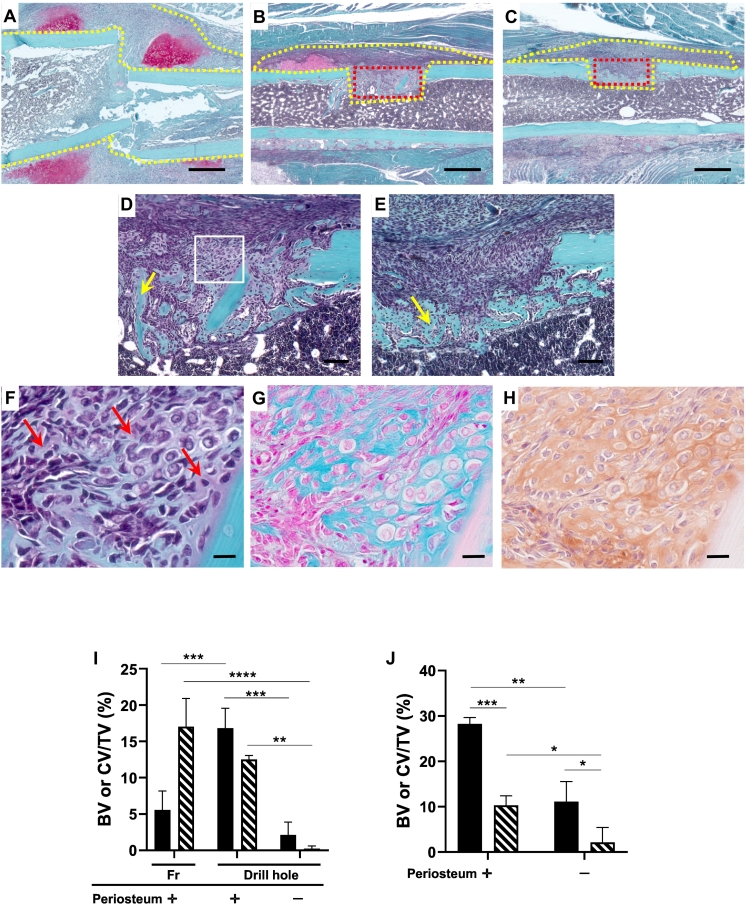
Histological comparison among intramedullary nailing femoral fracture (A), and drill hole model created in the cortical bone with (C, E) or without (B, D, F) removing the periosteum before making the drill holes on day 7 after the operation (Safranin O stain). Note that the cartilage matrix stained in Safranin O can be seen on the periosteum in A and B, but not in C. D is a higher magnification of gap region in B, and E is a higher magnification in gap region in C. Yellow arrows in D and E indicate newly formed bones. F, G, and H are higher magnification features surrounded by white square in D. In F, the red arrow indicates faintly stained areas with Safranin O. These areas are stained with alcian blue (G) and positive for aggrecan immunohistochemistry (H). Yellow dot lines in A, B, and C indicate ROI measured callus including cortical gap region, and red dot lines in B and C show ROI applied for measuring cortical gap. I: Percentages of bone volume (BV) or cartilage volume (CV)/tissue volume (TV) in intramedullary nailing femoral fracture (Fr) and drill hole injury with or without periosteum removal before the surgery. J: Percentages of bone volume (BV) or cartilage volume (CV)/tissue volume (TV) at the cortical gap area with or without periosteum removal before the surgery. *: *P*<0.05, **: *P*<0.01, ***: *P*<0.001, ****: *P*<0.0001. n = 3. Scale bar = 500 μm (A–C), 100 μm (D, E), and 50 μm (F, G, H).

We compared the differences in histopathological features among intramedullary nailing femoral fracture (A), and drill hole model created in the cortical bone with (C, E) or without (B, D, F) removing the periosteum before making the drill holes on day 7 after the operation. In the intramedullary nailing model, the fractures were repaired by a considerable amount of callus formation by endochondral ossification (Fig. 6A, I). The drill hole was repaired by intramembranous ossification associated with cartilage formation around the drill holes when the periosteum was retained before making the hole (Fig. 6B, D). In these cases, though the gap between the cortical bone was mostly repaired by intramembranous ossification, cartilage stained by Safranin O remained scattered in the trabecular bone (Fig. 6D, F). These areas are stained with alcian blue (G) and positive for aggrecan immunohistochemistry (H). In contrast, when drill holes were created after removal of the periosteum, bone defects were repaired by solely intramembranous ossification, lacking chondrocytes in one case (Fig. 6C, E). The other two cases were associated with a trace of cartilage among trabecular bone formed in cortical gap. Quantitative analyses of the ratio of the bone and cartilage area formed at the cortical gap revealed that the cartilage formation ratio, in cases of periosteal removal before making drill holes, significantly decreased compared to that in the cases without periosteal removal (Fig. 6).

## 4 Discussion

We compared the histopathological changes in healing processes between tooth extraction sockets and femoral fractures and showed that the tooth socket was repaired by intramembranous ossification and the femoral fracture of the intramedullary nailing model was healed by endochondral ossification. Immunohistochemical and RT-PCR analyses supported these findings.

In the present study, the expression of *Col2a1* mRNA of a cartilage-related gene showed significant marginal increased during the healing process of tooth extraction socket by RT-PCR analysis (Fig. 5A) and *in situ* hybridization (Supplemental Fig. 1), though histological and immunohistochemical studies revealed no cartilage cell appearance. Ting et al. (1993) first reported *Col2a1* mRNA expression in tooth extraction sockets by northern blot analysis and *in situ* hybridization. Other groups showed *Col2a1* mRNA expression during the healing process of tooth extraction sockets, associated with no cartilage formation (Devlin et al., 1997). Cheah et al. (1991) demonstrated that expression of the mouse *a1*(*II*) *collagen* gene is not restricted to cartilage during development. In addition, Nagao et al. (2016) reported that bone, meniscus, ligament, and synovium other than cartilage were target tissues in *Col2-cre*/*Col2-creER* mice. Although the distinct role of *Col2* expression during bone formation has not been well elucidated, Kuroda et al. (2021) recently reported that type II collagen is produced by a specific osteoblast subtype during auditory ossicle formation.

The appearance of endochondral ossification during bone regeneration is thought to be regulated by two major factors (Claes et al., 2012). First, cartilage appearance is affected by the mechanical environment; stable fixation induces intramembranous ossification and unstable fixation stimulates endochondral ossification (Claes et al., 2012; Vieira et al., 2015). Indeed, the tooth extraction socket always receives stable fixation surrounded by alveolar bone. In contrast, the intramedullary nailing model associated with interfragmentary movement evoked a large amount of endochondral ossification callus. Second, bone healing is influenced by revascularization in the healing region (Claes et al., 2012). When the injury sites are located away from the periosteum due to the interfragmentary distance, insufficient blood supply occurs. Such insufficient blood supply induces the low oxygen tension that disturbs osteoblast differentiation but allows chondrocyte proliferation and differentiation (Bassett and Herrmann, 1961; Miclau et al., 2017). The intramedullary nailing model of femoral fractures used in the present study adapted to these situations. This was further supported by a recent report by van Gastel et al. (2020); who revealed that skeletal progenitor cells in the bone regeneration area preferentially differentiate into chondrogenic cells rather than osteoblastic cells when blood supply is avoided by insertion of polycarbonate filters. Conversely, tooth extraction sockets might be suitable for retaining abundant hematomas at the injury site, which might support induction of well-developed revascularization. This situation might be favored to induce intramembranous bone formation. In addition, intimate coupling of type H endothelium and osteoprogenitors was reported to be essential for bone metabolism and bone healing (Kusumbe et al., 2014; Xu et al., 2018), and this coupling has also been observed in tooth extraction models in mice (Yan et al., 2020). Further investigation to compare type H endothelium distribution and function among different bone healing models is of interest to understand the role of type H endothelium in bone regeneration.

We showed that bone formation first occurred in regions adjacent to the alveolar bone surface in the healing process of tooth extraction socket, and it extended to the center of the socket. These findings indicate that osteoblastic cells participating in tooth socket healing were originated from the periodontium. Recently, lineage tracing studies offered the possibility to disclose the origin and fate of cells involved in bone healing, and such studies have been applied to explore cell lineages involved in tooth extraction healing; contrast *Axin2-CreERT2*-derived cells in bone injury of tibia (Ransom et al., 2016) with tooth extraction socket (Yuan et al., 2018)；contrast *Lepr-Cre*-derived mesenchymal cells in repair of semistabilized femoral fracture (Mizoguchi et al., 2014) with tooth extraction socket (Zhang et al., 2020)；contrast *Gli1-CreER*-derived stromal cells in healing of semistabilized femoral fracture (Shi et al., 2017) with tooth extraction socket (Yi et al., 2021). Interestingly, each *Cre*-marked cell in these mice participated in bone formation only during tooth socket healing, whereas they formed both cartilage and bone formation during healing process of long bone injury. These findings suggest that the plasticity of stem cells differs between tooth socket healing and long bone fractures.

Drill hole injury models of cortical bones created in long bone possess an environment similar to that of the tooth socket healing. The drill hole injury was retained by constant stability surrounded by cortical bone like alveolar bone in tooth extraction socket healing, and this environment provided stable retention of plasma clot necessary for the recruitment of revascularization. These similarities prompted us to compare histopathological changes between the healing processes of tooth socket and drill hole injury. We examined two types of drill hole models: with or without removal of periosteum before making drill holes. Both types of drill holes were repaired by mainly intramembranous ossification, whereas cartilage appeared in periosteal region adjacent to the bone defect when the periosteum was retained before making the drill hole, but few cartilages appeared adjacent to bone defects when the drill hole was created after extensive removal of the periosteum. These results suggest that the periosteum can differentiate into chondrocytes even under stable fixation and sufficient blood supply at periosteal region. Monfoulet et al. (2010) reported that drill hole injury created at the cortical bone of femur mid-diaphysis was repaired by intramembranous ossification in the absence of cartilage 14 days after the surgery regardless of the stripping of periosteum before drilling. In the present study, we observed a small amount of cartilage in the regenerating tissues that bridges the cortical gap 7 days after the surgery. All cases had retained periosteum before the operation exhibited a minute amount of cartilage. In the group that had the periosteum removed prior to the operation, two cases exhibited a trace amount of cartilage, while one case showed intramembranous ossification lacking cartilage. These differences from the previous report (Monfoulet et al., 2010) might be dependent on the time of observation after the operation; the previous report made observations 21 days after the operation, while we made observations 7 days after the operation. Alternatively, the cases exhibiting cartilage in the present study might be due to the incomplete removal of the periosteum because the periosteal stem cells can induce endochondral ossification in case of cortical defects (Debnath et al., 2018). Further, the stem cells at the cortical gap might retain bi-potential activity to differentiate into both osteoblast and chondrocyte lineage cells or facilitate cartilage to bone transformation (Hu et al., 2017). The cell lineage-tracing techniques applied to various regeneration steps can be employed to resolve this issue. Taken together, we presume that the plasticity of cartilage is different between periodontium and periosteum because tooth socket healing is never associated with cartilage formation. Further investigation is necessary to clarify whether the differences in plasticity between periodontium and periosteum might be caused by site-dependent manner, or intrinsic cellular characteristics, or both.

## 5 Conclusion

The present study demonstrated that the absence of cartilage appearance during tooth extraction socket healing indicates it as a distinct pathological feature of the healing processes of femoral fractures. This study establishes a research platform for understanding the molecular and cellular characteristics of tooth extraction socket healing based on pathological findings.

The following are the supplementary data related to this article.

Supplemental Fig. 1 *In situ* hybridization of Col2a1 mRNA. A: Tooth socket after day 7 of tooth extraction. Some cells show positive signals of Col2a1 mRNA in cytoplasm (blue arrow heads) by hybridization with Col2a1 antisense probe. B: Tooth socket after day 7 tooth extraction. No signal is observed by hybridization with Col2a1 sense probe. C: Tooth socket after day 7 of tooth extraction. Hybridized by 28S antisense probe. D: Cartilage callus of the semi-stabilized femur fracture after day 7. Chondrocytes show positive signals of Col2a1 mRNA. Scale bars represent 10 μm. Supplemental Fig. 1

Supplemental Table 1 The primer sequences used for each gene. Supplemental Table 1

## Funding

This research was supported by a grant for 10.13039/501100001700MEXT Private University Branding Project, and Branding Project for Multidisciplinary Research Center for Jaw Disease Fund, Tokyo Dental College. This work was partially supported by the 10.13039/501100001691Japan Society for the Promotion of Science KAKENHI, grant number 20H03894.

## CRediT authorship contribution statement

**Shinichirou Ito**: Conceptualization, Data curation, Formal analysis, Investigation, Methodology, Writing-original draft. **Norio Kasahara**; Formal analysis, Investigation, Methodology. **Kei Kitamura**; Investigation, Methodology. **Satoru Matsunaga**; Investigation, Methodology. **Toshihide Mizoguchi**; Conceptualization, Investigation, Methodology. **Myo Win Htun**; Methodology. **Yasuaki Shibata**; Methodology. **Shinichi Abe**; Conceptualization. **Masayuki Takano**; Conceptualization. **Akira Yamaguchi**; Conceptualization, Funding acquisition, Investigation, Supervision, Writing-review and editing. All authors read and approved the final manuscript.

## Declaration of competing interest

There were no financial or non-financial competing interests relevant to the published content. All authors declare no conflict of interests that might influence the result of this study.
