# Supplementary material for: Pathological differences in the bone healing processes between tooth extraction socket and femoral fracture
Source: Bone Rep. 2022 Mar 24;16:101522. doi: 10.1016/j.bonr.2022.101522 (PMC8965168; doi:10.1016/j.bonr.2022.101522)
Supplement: Supplemental Fig. 1 — In situ hybridization of Col2a1 mRNA. A: Tooth socket after day 7 of tooth extraction. Some cells show positive signals of Col2a1 mRNA in cytoplasm (blue arrow heads) by hybridization with Col2a1 antisense probe. B: Tooth socket after day 7 tooth extraction. No signal is observed by hybridization with Col2a1 sense probe. C: Tooth socket after day 7 of tooth extraction. Hybridized by 28S antisense probe. D: Cartilage callus of the semi-stabilized femur fracture after day 7. Chondrocytes show positive signals of Col2a1 mRNA. Scale bars represent 10 μm. [file mmc1.pptx]

## Slide 1
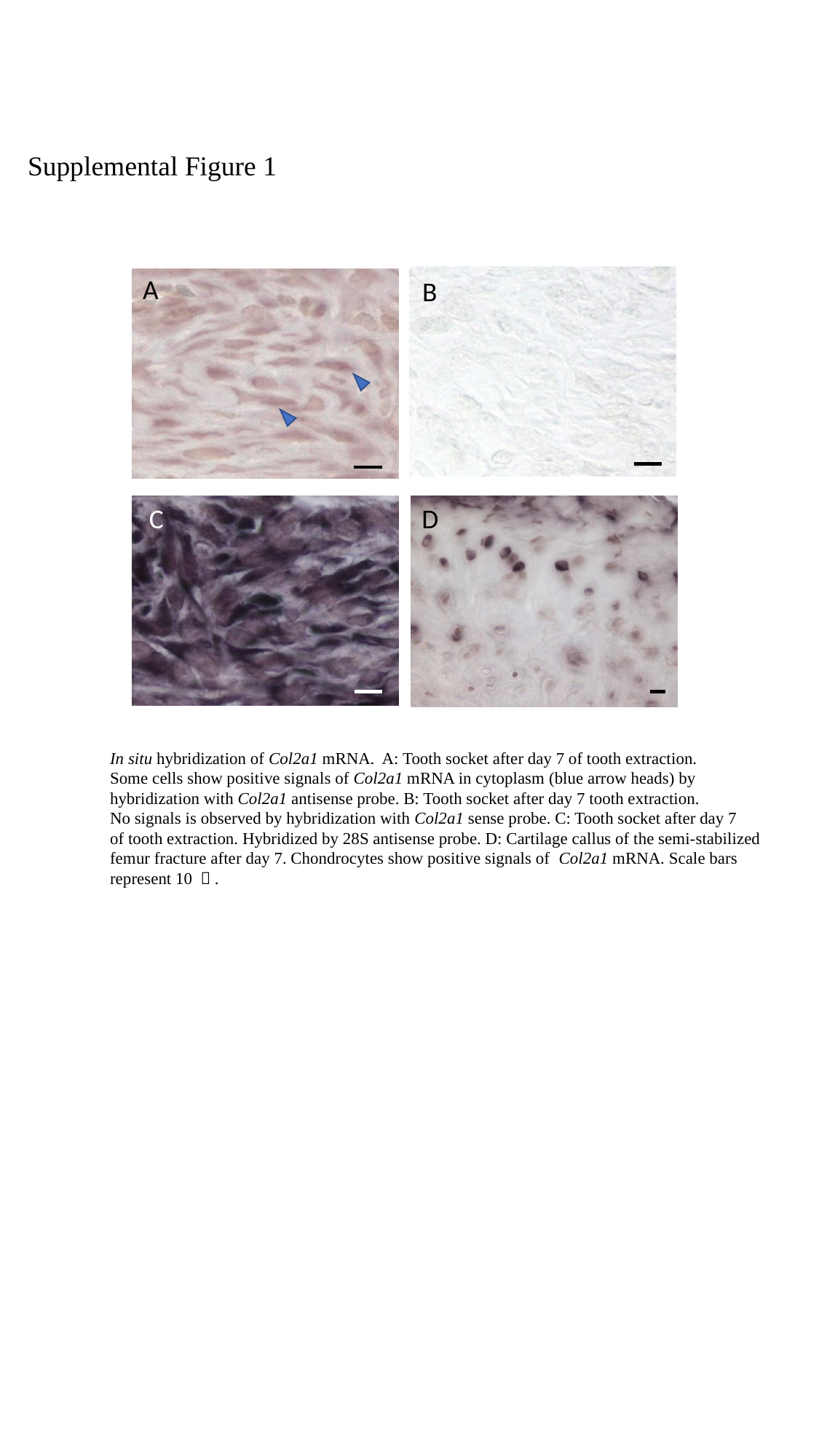

Supplemental Figure 1
A
B
 C
 D
In situ hybridization of Col2a1 mRNA. A: Tooth socket after day 7 of tooth extraction.
Some cells show positive signals of Col2a1 mRNA in cytoplasm (blue arrow heads) by
hybridization with Col2a1 antisense probe. B: Tooth socket after day 7 tooth extraction.
No signals is observed by hybridization with Col2a1 sense probe. C: Tooth socket after day 7
of tooth extraction. Hybridized by 28S antisense probe. D: Cartilage callus of the semi-stabilized
femur fracture after day 7. Chondrocytes show positive signals of Col2a1 mRNA. Scale bars
represent 10 ㎛.
