# Supplementary material for: Pathological differences in the bone healing processes between tooth extraction socket and femoral fracture
Source: Bone Rep. 2022 Mar 24;16:101522. doi: 10.1016/j.bonr.2022.101522 (PMC8965168; doi:10.1016/j.bonr.2022.101522)
Supplement: Supplemental Table 1 — The primer sequences used for each gene. [file mmc2.pptx]

## Slide 1
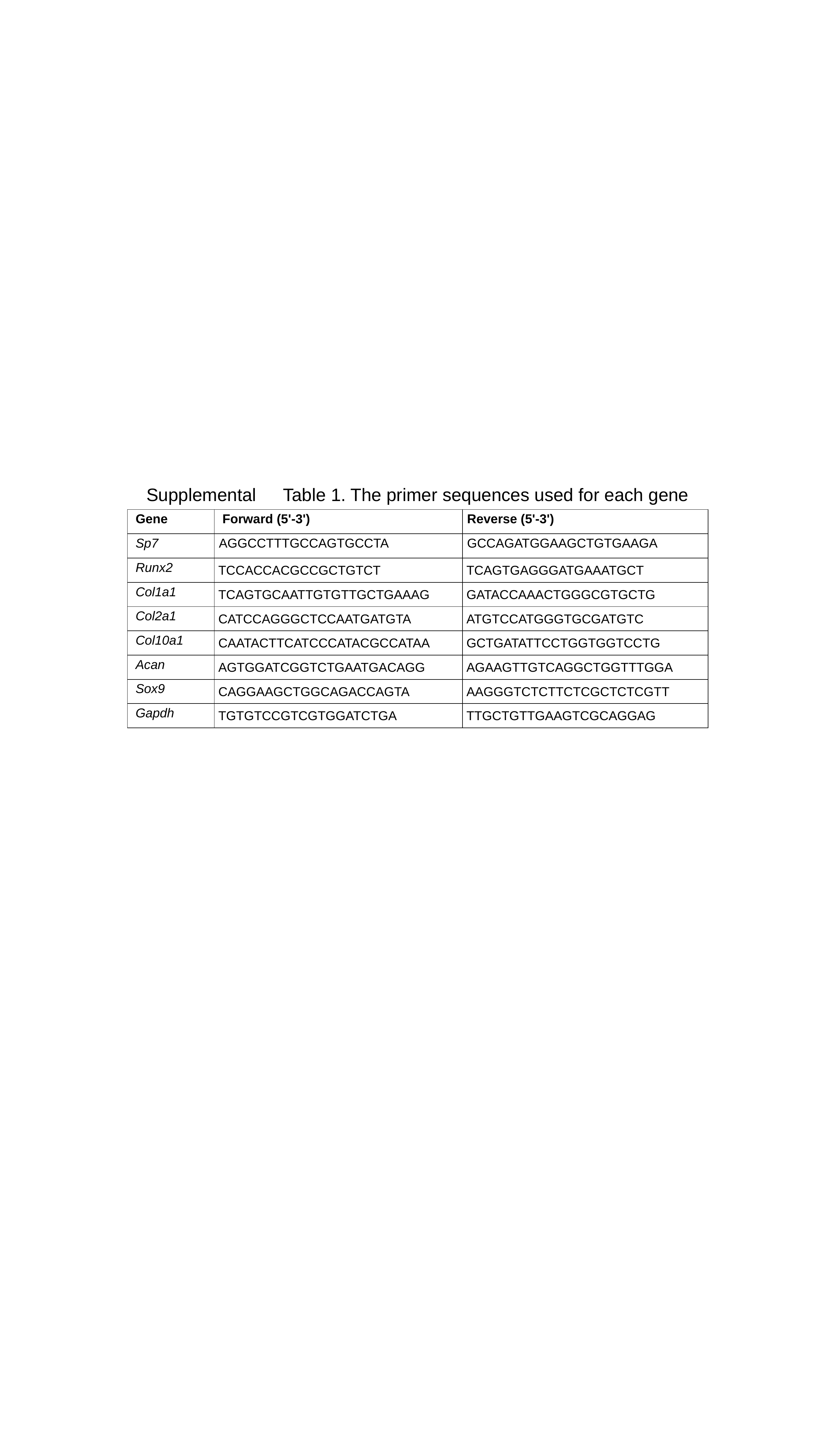

Supplemental　Table 1. The primer sequences used for each gene
| Gene | Forward (5'-3') | Reverse (5'-3') |
| --- | --- | --- |
| Sp7 | AGGCCTTTGCCAGTGCCTA | GCCAGATGGAAGCTGTGAAGA |
| Runx2 | TCCACCACGCCGCTGTCT | TCAGTGAGGGATGAAATGCT |
| Col1a1 | TCAGTGCAATTGTGTTGCTGAAAG | GATACCAAACTGGGCGTGCTG |
| Col2a1 | CATCCAGGGCTCCAATGATGTA | ATGTCCATGGGTGCGATGTC |
| Col10a1 | CAATACTTCATCCCATACGCCATAA | GCTGATATTCCTGGTGGTCCTG |
| Acan | AGTGGATCGGTCTGAATGACAGG | AGAAGTTGTCAGGCTGGTTTGGA |
| Sox9 | CAGGAAGCTGGCAGACCAGTA | AAGGGTCTCTTCTCGCTCTCGTT |
| Gapdh | TGTGTCCGTCGTGGATCTGA | TTGCTGTTGAAGTCGCAGGAG |
